# A multi-country study of the associations between HIV vulnerability status, perception of COVID-19 related stigma and post-traumatic stress symptoms during the first wave of the pandemic

**DOI:** 10.1186/s12889-023-15933-z

**Published:** 2023-05-30

**Authors:** Morenike Oluwatoyin Folayan, Roberto Ariel Abeldaño Zuñiga, Jorma I. Virtanen, Passent Ellakany, Ala’a B. Al-Tammemi, Mir Faeq Ali Quadri, Mohammed Jafer, Eshrat Ara, Martin Amogre Ayanore, Balgis Gaffar, Nourhan M. Aly, Ifeoma Idigbe, Joanne Lusher, Oliver C. Ezechi, Annie L Nguyen, Maha El Tantawi

**Affiliations:** 1 Mental Health and Wellness Study Group, Ile-Ife, Nigeria; 2 Department of Child Dental Health, Obafemi Awolowo University, Ile-Ife, Nigeria; 3 Postgraduate Department, University of Sierra Sur, Oaxaca, Mexico; 4 Faculty of Social Sciences, University of Helsinki, Helsinki, Finland; 5 Faculty of Medicine, University of Turku, Turku, Finland; 6 Department of Substitutive Dental Sciences, College of Dentistry, Imam Abdulrahman Bin Faisal University, Dammam, Saudi Arabia; 7 Applied Science Research Center, Applied Science Private University, Amman, Jordan; 8 Migration Health Division, International Organization for Migration, Amman, Jordan; 9 Department of Oral Health Sciences, University of Washington, Washington, USA; 10 Dental Public Health Division, Faculty of Dentistry, Jazan University, Jizan, Saudi Arabia; 11 Department of Psychology, Government College for Women, MA Road, Jizan, J&K India; 12 Department of Health Policy Planning and Management, Fred N. Binka School of Public Health, University of Health and Allied Sciences, Ho, Ghana; 13 Department of Preventive Dental Sciences, College of Dentistry, Imam Abdulrahman bin Faisal University, Dammam, Saudi Arabia; 14 Department of Pediatric Dentistry and Dental Public Health, Faculty of Dentistry, Alexandria University, Alexandria, Egypt; 15 Clinical Sciences Department, Nigerian Institute of Medical Research, Lagos, Nigeria; 16 Provosts Group, Regent’s University London, London, UK; 17 Centre for Reproductive and Population Health Studies, Nigerian Institute of Medical Research Yaba, Lagos, Nigeria; 18 Department of Family Medicine, Keck School of Medicine, University of Southern California, Los Angeles, CA USA

**Keywords:** COVID-19, HIV, Stress disorders, Post-traumatic, Social stigma

## Abstract

**Background:**

This study investigated the associations between COVID-19 related stigma and post-traumatic stress symptoms (PTSS); and the associations between PTSS and COVID-19 related stigma, HIV status, COVID-19 status and key HIV population status.

**Methods:**

This was a secondary analysis of data of 12,355 study participants generated through an online survey that recruited adults from 152 countries between July and December 2020. The dependent variables were COVID-19-related stigma and PTSS. The independent variables were HIV status (positive/negative), transaction sex (yes/no), use of psychoactive drugs (yes/no), and vulnerability status (transaction sex workers, people who use psychoactive drugs, living with HIV, and COVID-19 status). The confounding variables were age, sex at birth (male/female), level of education, sexual minority individuals (yes/no) and country income level. Multivariable logistic regression analyses were conducted to determine associations between the dependent and independent variables after adjusting for confounders.

**Results:**

There were 835 (6.8%) participants who experienced COVID-19 related stigma during the pandemic and 3,824 (31.0%) participants reported PTSS. Respondents who were living with HIV (AOR: 1.979; 95%CI: 1.522–2.573), tested positive for COVID-19 (AOR: 3.369; 95%CI: 2.692–4.217), engaged in transactional sex (AOR: 1.428; 95%CI: 1.060–1.922) and used psychoactive drugs (AOR: 1.364; 95%CI: 1.053–1.767) had significantly higher odds of experiencing COVID-19 related stigma. Individuals with vulnerability status (AOR:4.610; 95%CI: 1.590-13.368) and who experienced COVID-19 related stigma (AOR: 2.218; 95%CI: 1.920–2.561) had significantly higher odds of PTSS.

**Conclusion:**

Individuals with vulnerability status may be at increased risk for COVID-19 related stigma. Key and vulnerable populations who were living with HIV and who experienced stigma may be at a higher risk of experiencing PTSS. Populations at risk for PTSS should be routinely screened and provided adequate support when they contract COVID-19 to reduce the risk for poor mental health during COVID-19 outbreaks and during future health crisis with similar magnitude as the COVID-19 pandemic.

## Introduction

Many individuals and their families who recovered from COVID-19 have faced double challenges- the physical health fallout from experiencing COVID-19 symptoms and related complications, and the COVID-19 related stigma that is driven by fear and misconception about the disease [1, 2]. Stigma is the disapproval of or negative attitudes toward persons with certain characteristics or diseases that distinguish them from other members of society [3]. It is either enacted or self, and results from stereotypes, labelling, prejudice, and discrimination learned by most members of a social group [4–6].

One of the potential consequences to experiencing stigma is depression [7, 8]. Depression and stigma share common symptoms such as guilt feeling, low self-esteem, and self-blame [7, 8]. Social exclusion, rejection, eviction from homes of residence, insult, and the burden of responsibility to reduce family and friends’ risk of contracting the COVID-19, create mental health challenges for COVID-19 affected persons [9–12]. People who experience stigma may also experience discrimination, psychological stress, and suicide ideation [13]. Moreover, stigma constitutes a major barrier to the containing COVID-19 pandemic because it perpetuates fears of contracting the virus, poor knowledge about the transmission of the disease, and concerns about dying from the disease [14]. As a result, stigma makes the people less willing to test for the virus, to be diagnosed, or even feel reluctant to seek treatment when they are infected [9, 15–17].

There are many individuals who may feel more vulnerable to the consequences of COVID-19 associated stigma. People living with HIV, sexual minority individuals and key populations in the HIV response (people who inject drugs, people who transact sex, men who have sex with men, transgender people, and people in prisons and closed settings [18]) face stigma and discrimination more often than members of the general population [19–25]. HIV-positive individuals are vulnerable to stigma just like COVID-19 [26]. Like COVID-19, HIV is infectious with no definite therapy. They are both associated with avoidance, blame, and discrimination [27] as identified by people living with HIV who also had COVID-19 [28]. Both forms of stigma are associated with mental health challenges [29, 30]. For individuals who consistently experience social and disease-related stigma, the risk of experiencing COVID-19 related stigma may be a déjà vu experience.

In the present study, we utilized the Framework Integrating Normative Influences on Stigma (FINIS) as the conceptual model to facilitate understanding stigma experiences [31]. The framework articulates that the stigmatization process can be broken down into a series of constituent domains, including drivers, facilitators, stigma ‘marking’, and stigma manifestations, which influence a range of outcomes among affected populations that ultimately impact health and society [32]. We conceptualise that the fear of SARS-CoV-2 infection may be a driver of COVID-19 related stigma [1, 2]. However, there are norms, legal environments and policies that facilitate the enactment of health-condition (in this study we looked at HIV and COVID-19) and sexual orientation related stigma; and the perception or experiencing of COVID-19 related stigma. This may cause post traumatic stress symptoms (PTSS).

The Framework recognises that both social and illness characteristics shape the experience of stigma. Additionally, social and illness characteristics combined to shape the experience of stigma, and different levels of social life influence the experience of stigma [33]. We postulate that people who had past experiences of stigma due to social and illness characteristics may be more likely to experience COVID-19 related stigma especially people whose way of life may not conform with normative expectations [32]. These include people living with HIV, who may be extremely cautious during the pandemic because of the fear of severe infection [34, 35]; people injecting drugs due to increased risk of relapse [36]; sex workers who may experience increased dependency due to loss of income [37]; and sexual minority individuals due to stigmatization of decisions to engage in sexual behaviours during a time of mandated quarantines [38]. We also postulate that the experience of stigma may be confounded by the level of economic development of the society.

In addition, though people living with HIV, sexual minority individuals and key populations have a comparable biological risk profile for COVID-19 to that of the general population [39–43], their experience of COVID-19 related stigma may differ. A prior experience of negative labelling and social exclusion may amplify the risk of COVID-19 related stigma. For those who experience multiple forms of stigma, the intersectional of those stigmas – health (COVID-19, HIV), sexual orientation (sexual minority individuals), occupation (sex work) and lifestyle (drug use) – could increase the risk for post-traumatic stress disorder (a disorder that develops in some people who experience a shocking, scary, or dangerous event) [44–46].

The aims of this study, therefore, were to assess associations between reported experience of COVID-19 pandemic related stigma and living with HIV and key HIV population status; and assess the association between post-traumatic stress symptoms and the self-reported experience of COVID-19 pandemic stigma. We hypothesised that (1) people living with HIV and being a member of a key population will be positively associated with the experience of COVID-19 stigma and (2) the experience of COVID-19 stigma will be positively associated with post-traumatic stress disorder.

## Methods

### Ethical considerations

Ethical approval was obtained from the Human Research Ethics Committee at the Institute of Public Health of the Obafemi Awolowo University Ile-Ife, Nigeria (HREC No: IPHOAU/12/1557). Ethical approval was also obtained from Brazil (CAAE N° 38423820.2.0000.0010), India (D-1791-uz and D-1790-uz), Saudi Arabia (CODJU-2006 F) and the United Kingdom (13,283/10,570) for the conduct of the primary study. Study participants checked a box to indicate consent before participating in the online survey.

### Study design and study population

This was a secondary analysis of data generated through a cross-sectional international study that recruited adults from 152 countries through an online survey between July and December 2020 during the first wave of the COVID-19 pandemic. Study participation was open to anyone 18 years and older. There were no exclusion criteria.

### Sample size

The sample size for the primary study was calculated based on the highest global prevalence of a mental health disorder in 2019. The pre-survey minimum sample size for this study was set at 59 valid respondents from each of the 193 member States of the United Nations based on the estimated prevalence of the most common global mental health disorder in 2019, (3.94% for anxiety disorder) [47]; a desired precision of estimate was 0.05 and a confidence level was 95% for an infinite population size [48]; and increased by 10% to accommodate non response and incomplete responses [49]. From the statistical modelling perspective, a minimum of 10 participants with complete responses per each of the independent variables enabled the performance of regression analyses with a minimum probability level (p-value) of 0.05 [50]. For this study, the data of 12,355 (58.5%) respondents with complete responses were extracted from the 21,106 respondents.

### Participant recruitment

Study participants were recruited through respondent-driven sampling. Initially, 45 members of the mental health and wellness (MEHEWE) study group (www.mehewe.org) were asked to share the survey link with their contacts around the world to facilitate recruitment. The survey link was also posted on social media groups (Facebook, Twitter, and Instagram), network email lists and WhatsApp groups. The details of how the survey was conducted and the data collection tools are published elsewhere [51–55].

### Data collection tool

Quantitative and qualitative assessments were conducted to validate the study questionnaire. The overall content validity index for the study questionnaire was 0.83. The dimensionality and reliability of the tool were also assessed. Qualitative analysis showed that participants agreed that the multidimensional assessment captured the effect of the pandemic though it was better suited for those with a high level of education. The details on the validation of the tool for the data collection had also been published [51]. The instrument was developed in English and the validated instrument translated into French, Spanish, Arabic and Portuguese.

### Dependent variables

There were two dependent variables: reported experience of COVID-19 related stigma or discrimination and post-traumatic stress symptoms (a proxy measure for post-traumatic stress disorder [56]). For COVID-19 related stigma, participants were asked to check a box if they had experienced stigma or discrimination from other people (people treating you differently because of your identity, having symptoms, or other factors related to COVID-19) during this COVID-19 period. Those who checked the box were categorised as having experienced COVID-19 related stigma or discrimination.

Post-traumatic stress symptoms (PTSS) were measured using the 17-item self-report PTSD checklist for civilians [57]. This is a 5-point scale with responses ranging from 1- “not at all” to 5- “extremely”. The possible total score ranged from 17 to 85 with a cut-off of 28 used to dichotomise the responses to “no PTSS” (17–27) and “PTSS present” (28–85) [58]. The details of the measure had been reported in two prior studies published from the same data set [54, 55]. The Cronbach alpha for the PTSD items was 0.93, the test-retest validation score was 0.83, the content validation index was 0.81 and the intra-class correlation coefficient was 0.89 indicating excellent reliability [55]. For this study, the Cronbach alpha was 0.96.

### Independent variables

The independent variables were the subpopulations previously identified. These are: HIV key populations (people who engaged in transactional sex, people who use psychoactive drugs) vulnerable to social stigma [59, 60], people living with HIV [61], and people who had COVID-19 [62]. Respondents identified their status by ticking a box. All respondents who ticked the checkbox were designated as having a vulnerability status.

### Confounding variables

The confounding variables were age at last birthday, sex at birth (male, female), sexual identity (heterosexual, sexual minority individuals, undisclosed), level of education (no formal education, primary, secondary and college/university) and country of resident’s income level (low-income countries (LICs), lower middle-income countries (LMICs), upper middle-income countries (UMICs) and high-income countries (HICs). Respondents had to pick a country of residence from a list. The country was them categorised into the country income level using the 2019 World Bank Data on country classification by Gross National Income [63].

### Data analysis

Raw data were downloaded, cleaned, and imported to SPSS version 23.0 (IBM Corp., Armonk, N.Y., USA) for analysis. A multivariable logistic regression analysis was conducted to determine the associations between reported experience of COVID-19 related stigma and the independent variables (living with HIV, had COVID-19, use of psychoactive drugs and engaging in transactional sex) after adjusting for confounders. A second multivariable logistic regression analysis was conducted to determine the associations between PTSS and the independent variables (experience of COVID-19 related stigma, vulnerability status) after adjusting for confounders. Adjusted odds ratios (AOR), 95% confidence intervals (CI), and p values were calculated. Statistical significance was set at 0.05.

## Results

Of the 12,355 respondents whose data were extracted for this study, 835 (6.8%) experienced COVID-19 related stigma. Table 1 shows that respondents living with HIV (AOR: 1.979; 95%CI: 1.522–2.573) and those who had COVID-19 (AOR: 3.369; 95%CI: 2.692–4.217) had significantly higher odds of experiencing stigma than people not living with HIV and those did not have COVID-19 respectively. Also, respondents who engaged in transactional sex (AOR: 1.428, 95%CI: 1.060–1.922) and those who used psychoactive drugs (AOR: 1.364; 95%CI: 1.053–1.767) had significantly higher odds of experiencing COVID-19 related stigma than respondents who did not engage in transactional sex or used psychoactive drugs respectively.

**Table 1:** Multivariable logistic regression analysis for the associations between reported experience of COVID-19 related stigma or discrimination, HIV status, COVID-19 status, use of psychoactive drugs and engaging in transactional sex during the first wave of the pandemic (N = 12,355)

Variables	Total N = 12,355 n (%)	Experienced COVID-19 related stigma		AOR; 95% CI; p value
		No N = 11,520 (93.2%) n (%)	Yes N = 835 (6.8%) n (%)	
Living with HIV* Yes No	905 (7.3) 11,450 (92.7)	817 (90.3) 10,703 (93.5)	88 (9.7) 747 (6.5)	1.979; 1.522–2.573; p < 0.001 1.000
Had COVID-19* Yes No	625 (5.1) 11,730 (94.9)	505 (80.8) 11,015 (93.9)	120 (19.2) 715 (6.1)	3.369; 2.692–4.217; p < 0.001 1.000
Engage in transactional sex * Yes No	452 (3.7) 11,903 (96.3)	384 (85.0) 11,136 (93.6)	68 (15.0) 767 (6.4)	1.428; 1.060–1.922; p = 0.019 1.000
Use psychoactive drugs* **Yes** No	722 (5.8) 11,633 (94.2)	640 (88.6) 10,880 (93.5)	82 (11.4) 753 (6.5)	1.364; 1.053–1.767; p = 0.019 1.000
**Mean age (Standard deviation)**	35.8 (13.1)	36.0 (13.2)	33.4 (11.4)	0.979; 0.973–0.985; p < 0.001
**Sex at birth** Male Female	4487 (36.3) 7868 (63.7)	4135 (92.2) 7385 (93.9)	352 (7.8) 483 (6.1)	1.000 0.804; 0.693–0.932; p = 0.004
**Sexual identity** Heterosexual Sexual minority individuals Undisclosed	9655 (78.1) 1146 (9.3) 1554 (12.6)	8999 (93.2) 1033 (90.1) 1488 (95.8)	656 (6.8) 113 (9.9) 66 (4.2)	1.000 1.145; 0.913–1.437; p = 0.242 0.660; 0.505–0.863; p = 0.002
**Educational status** No formal education Primary Secondary College/University	291 (2.4) 352 (2.8) 2282 (18.5) 9430 (76.3)	283 (97.3) 338 (96.0) 2120 (92.9) 8779 (93.1)	8 (2.7) 14 (4.0) 162 (7.1) 651 (6.9)	0.397; 0.194–0.815; p = 0.012 0.512; 0.295–0.888; p = 0.017 0.932; 0.773–1.123; p = 0.459 1.000
**Country income level** LICs LMICs UMICs HICs	189 (1.5) 6983 (56.5) 2484 (20.1) 2699 (21.8)	175 (92.6) 6556 (93.9) 2317 (93.3) 2472 (92.6)	14 (7.4) 427 (6.1) 167 (6.7) 227 (8.4)	0.899; 0.529–1.585; p = 0.712 0.675; 0.562–0.810; p < 0.001 0.854; 0.690–1.057; p = 0.147 1.000
Nagelkerke R2 0.052				
Omnibus test of model coefficients Chi-square = 257.641; p < 0.001				

*Adjusted for: age, sex at birth, sexual identity, educational status and country income level

Table 2 shows that there were 3,824 (31.0%) respondents who experienced PTSS during the COVID-19 pandemic. Respondents with vulnerability status had significantly higher odds of PTSS than the respondents who were not vulnerable (AOR: 4.610; 95%CI: 1.590-13.368). In addition, those who experienced COVID-19 related stigma had significantly higher odds of experiencing PTSS than those who did not (AOR: 2.218; 95%CI: 1.920–2.561).

**Table 2:** Multivariable logistic regression analysis for the associations between the COVID-19 pandemic induced post-traumatic stress symptoms, reported COVID-19 related stigma vulnerability status during the first wave of the pandemic (N = 12,355)

Variables	Total N = 12,355 (100%) n (%)	Post-traumatic stress symptoms		AOR; 95% CI; p value
		Yes N = 3824 (31.0%) n (%)	No N = 8531 (69.0%) n (%)	
Experienced stigma and discrimination* Yes No	835 (6.8) 11,520 (93.2)	397 (47.5) 3427 (29.7)	438 (52.5) 8093 (70.3)	2.218; 1.920–2.561; p < 0.001 1.000
Vulnerable* Yes No	18 (0.1) 12,337 (99.9)	13 (72.2) 3811 (30.9)	5 (27.8) 8526 (69.1)	4.610; 1.590-13.368; p = 0.005 1.000
**Mean age (Standard deviation)**	35.8 (13.1)	31.9 (11.3)	37.6 (13.5)	0.967; 0.963–0.970; p < 0.001
**Sex at birth** Male Female	4487 (36.3) 7868 (63.7)	1152 (25.7) 2672 (34.0)	3335 (74.3) 5196 (66.0)	1.000 1.403; 1.287–1.530; p < 0.001
**Educational status** No formal education Primary Secondary College/University	291 (2.4) 352 (2.8) 2282 (18.5) 9430 (76.3)	231 (79.4) 205 (58.2) 905 (39.7) 2483 (26.3)	60 (20.6) 147 (41.8) 1377 (60.3) 6947 (73.7)	10.984; 8.156–14.791; p < 0.001 4.150; 3.311–5.202; p < 0.001 1.550; 1.402–1.713; p < 0.001 1.000
**Country income level** LICs LMICs UMICs HICs	189 (1.5) 6983 (56.5) 2484 (20.1) 2699 (21.8)	51 (27.0) 2334 (33.4) 643 (25.9) 796 (29.5)	138 (73.0) 4649 (66.6) 1841 (74.1) 1903 (70.5)	1.051; 0.747–1.477; p = 0.776 0.898; 0.810–0.997; p = 0.043 0.824; 0.726–0.936; p = 0.003 1.000
Nagelkerke R2: 0.132				
Omnibus test of model coefficients Chi-square = 1213.907; p < 0.001				

*Adjusted for: age, sex at birth, educational status and country income level

## Discussion

The study findings suggest that populations from groups that have traditionally experienced stigma and discrimination (people who use psychoactive drugs, transactional sex workers and people living with HIV) were more likely to experience COVID-19 related stigma. Also, people who experienced COVID-19 related stigma were more likely to report PTSS than those who did not. Individuals who were vulnerable to social stigma had higher risks of PTSS. Thus, the findings suggest that groups of people who were already at risk of experiencing stigma may be more vulnerable to experiencing COVID-19 related stigma and the challenges that come with this addition stigma such as PTSS. The results of this study support the study hypotheses.

This is one of few studies to provide empirical evidence on the possible risks for experiencing COVID-19 related stigma, and the effects of this on the mental health of affected individuals. Stigma can affect individual’s mental health negatively; and marginalized groups, including sexual minority individuals, individuals with physical diseases and socially excluded individuals, have a higher risk of experiencing stigma [64, 65].

The study has some limitations. This was a cross-sectional study which makes it difficult to establish a cause-effect relationship between the variables. Also, a non-probability sampling technique was used to recruit the study population. While this is the most appropriate way to recruit hidden populations like those identified for this study [66–68], and also an appropriate method for data collection during the COVID-19 pandemic [69, 70], the use of online platforms for data collection may have inadvertently excluded populations who are not able to access the internet and smartphones or similar devices. The study also excluded those without access to the survey in their languages. These limitations the generalisability of study findings to those who are potentially more vulnerable such as individuals with lower educational attainment and socioeconomic status. The results reported here may therefore, underrepresent the true magnitude of the problem. Also, because PTSS is self-reported, prevalence of post-traumatic stress disorder may be overestimated [71]. In addition, the experience of stigma was measured by a single question. While many consider single-item measures are an unsound approach to measuring cognitive and affective outcomes, single-item measures can provide valid and reliable assessments of important psychological phenomena just like their multi-item counterparts [72]. Finally, stigma related to HIV, sex work, and drug use were not measured so we are unable to characterize whether participants experienced stigmas in these domains. For these reasons, the study findings need to be interpreted with caution. Despite these limitations, the findings provide valuable evidence for policy and programmatic considerations. The findings can also assist in developing further hypotheses.

First, the study findings showed that populations that more likely to experience stigma and discrimination were also more likely to experience COVID-19 related stigma. Membership in stigmatised groups is known to amplify the risk of the stigma associated with health problems [33]. This is part of a social process that excludes those who are perceived to deviate from the acceptable standards in a sociocultural framework [73, 74]. The social identity theory further explains this phenomenon by noting that individuals think of themselves as individuals or as group members through a process of social categorization and social identification. As group members, individuals then want to maintain a positive social identity by maintaining their group’s favourable social standing over that of out-groups through social comparison. Thus, people stigmatise others who do not belong to their group because it gives stigmatisers an elated sense of self-esteem [75]. The findings also show that living with HIV and who had COVID-19 were significantly and independently associated with experiencing stigma. This implies that populations at risk of being stigmatised may not need to have tested positive for COVID-19 to experience COVID-19 related stigma. This has been demonstrated in research that showed some people who were not infected with COVID-19 were labelled as being infected with COVID-19 [76]. There is also the possibility of COVID-19 related self-stigma among people who had previous experience of stigma. These individuals may have increased activation of the amygdala and insula related to aversive emotions, and the anterior cingulate and lateral prefrontal cortex associated with cognitive control [77] leading to greater chances of self-stigmatisation. These possibilities need to be studied further.

The study findings also suggest that experiencing COVID-19 related stigma and discrimination may increase the risk of PTSS. This risk is about five times greater for people living with HIV and those who tested positive for COVID-19. Thus, being infected with COVID-19 exposes key populations living with HIV to the risk of a third stigma. Our finding agrees with previous research showing that stigma is a risk factor for post-traumatic stress disorder [78, 79]. In addition, we provide new evidence that experiencing COVID-19 related stigma and discrimination, and the experience of COVID-19 related stigma and discrimination by persons who have risk of past experiences of stigma because their lifestyle or from living with HIV, may significantly increases the risk for post-traumatic stress disorder. Therefore, populations vulnerable to stigma who contract COVID-19 would benefit from mental health support to reduce their risk of post-traumatic stress disorder.

It is recognised that COVID-19 stigma results from a complex web of expectations caused by individual and contextual interactions between the stigmatised and the stigmatisers. The theoretical framework that drives this study (FINIS) attempts to explain the community and individual factors that drives stigma. It recognises that the risk of stigma is higher when persons who hold devalued statuses are aware of this devalued social identity as this influences the perception and response to the social slights of stigma and acts of discrimination [80]. Key populations, sexual minority individuals and people living with HIV face devalued social status and thus, are more likely to respond to social cues of stigma associated with COVID-19. The negative social cues associated with COVID-19 were reinforced by public messages of COVID-19 infection danger appraisal [81]. This increases the possibility of COVID-19 related stigma being a global phenomenon. However, this phenomenon can be addressed by societal contexts emphasizing the acceptability of acting on cultural biases [33]. Though FINIS was developed to appraise the experience of stigma and discrimination by people with mental illness, we find the framework applicable to the experience of COVID-19 related stigma.

Furthermore, we observed that individuals who do not disclose their sexual identity seem to be protected from COVID-19 related stigma. Though we handled sexual identity as a confounding variable, the study finding suggests that this variable may moderate the reporting of stigma. Prior studies identified that sexual minority individuals avoid disclosing their sexual identity in real life to reduce being stigmatized [82–85]. The concealment of sexual identity doing research may however, not imply concealment in real life. Further research is needed to understand the reasons for concealment of sexual identity for research purposes, to enable appropriate interpretation of study findings.

Our study confounding variables - age, educational status, sex at birth and the country of residence - may also moderate the experience of stigma and PTSS. We observed that the likelihood of experience COVID-19 related stigma and PTSS decreased with age, as reported in previous studies [86–89]. The association between educational status and PTSS reported in this study had also been reported elsewhere [90, 91]. Also, we observed that females were less likely to experience COVID-19 related stigma and discrimination as reported in previous studies [92]. We also observed that although females were less likely to experience COVID-19 related stigma, which may be related to the fact that more males than females test positive to COVID-19 [92], more females experience PTSS as reported in past studies [93–96]. This paradox may suggest that females are better able to cope with stigma but are less able to deal with other factors that predispose to PTSS during the COVID-19 pandemic. This observed paradox needs to be studied further.

In conclusion, populations at risk of experiencing stigma prior to the pandemic such as people who inject drugs, transactional sex workers, sexual minority individuals and people living with HIV, were at higher risk of experiencing COVID-19 related stigma. The risk of PTSS may be higher for people who face COVID-19 related stigmatization and those with combined HIV and COVID-19 infection. People living with HIV, key populations and sexual minority individuals may need to be screened for PTSD when accessing health care services to facilitate prompt management. The study suggests a complex relationship with clustering of various stigmas that may have been deepened by the COVID-19 pandemic.

## Data Availability

The datasets used and/or analysed during the current study are available from the corresponding author upon reasonable request.

## Abbreviations

AOR: Adjusted Odds Ratio
CI: Confidence Interval
COVID-19: Coronavirus infectious disease 2019
HIV: Human Immunodeficiency Virus
SARS-CoV-2: Severe Acute Respiratory Syndrome Coronavirus 2
SD: Standard Deviation

## References

[1] Chew CC, Lim XJ, Chang CT (2021). Experiences of social stigma among patients tested positive for COVID-19 and their family members: a qualitative study. BMC Public Health.

[2] Williams J, Gonzalez-Medina D (2011). Infectious diseases and social stigma. Appl Innovations Technol.

[3] Williams J, Gonzalez-Medina D (2011). Infectious diseases and social stigma. Appl Innovations Technol.

[4] Hilton J, von Hippel W (1996). Stereotypes Annu Rev Psychol.

[5] Judd C, Park B (1996). Definition and assessment of accuracy in stereotypes. Psychol Rev.

[6] Krueger J (1996). Personal beliefs and cultural stereotypes about racial characteristics. J Pers Soc Psychol.

[7] Roeloffs C, Sherbourne C, Unützer J, Fink A, Tang L, Wells KB (2003). Stigma and depression among primary care patients. Gen Hosp Psychiatry.

[8] Yuan Y, Zhao YJ, Zhang QE (2021). COVID-19-related stigma and its sociodemographic correlates: a comparative study. Global Health.

[9] Adom D, Adu Mensah J (2020). The psychological distress and mental health disorders from COVID-19 stigmatization in Ghana. Social Sci Humanit.

[10] Bu N-N. The 80 thousand COVID-19 survivors are undergoing discrimination (in chinese). 2020. http://k.sina.com.cn/article_1690367810_64c0f74201900py8d.html?from=mood (access 18 July 2020).

[11] Do Duy C, Nong VM, Van AN, Thu TD, Do Thu N, Quang TN (2020). COVID-19 related stigma and its association with mental health of health-care workers after quarantined in Vietnam. Psychiatry Clin Neurosci.

[12] Duan W, Bu H, Chen Z (2020). COVID-19-related stigma profiles and risk factors among people who are at high risk of contagion. Soc Sci Med.

[13] Shoib S, Nagendrappa S, Grigo O, Rehman S, Ransing R (2020). Factors associated with COVID-19 outbreak-related suicides in India. Asian J Psychiatr.

[14] National Center for Chronic Disease Prevention and Health Promotion, Division of Population Health. Reducing Stigma. July 22., 2021 Available from: https://www.cdc.gov/mentalhealth/stress-coping/reduce-stigma/index.html. Accessed: 11th November, 2022.

[15] Grover S, Singh P, Sahoo S, Mehra A (2020). Stigma related to COVID-19 infection: are the health care workers stigmatizing their own colleagues?. Asian J Psychiatr.

[16] Earnshaw VA, Brousseau NM, Hill EC, Kalichman SC, Eaton LA, Fox AB (2020). Anticipated stigma, stereotypes, and COVID-19 testing. Stigma Health.

[17] World Health Organization. Consolidated Guidelines on HIV prevention, diagnosis, treatment and care for key populations. Geneva: 2014. [cited 2020 Apr 8]. Available from: https://www.who.int/hiv/pub/guidelines/keypopulations/en/. 25996019

[18] Akour A, AlMuhaissen SA, Nusair MB (2021). The untold story of the COVID-19 pandemic: perceptions and views towards social stigma and bullying in the shadow of COVID-19 illness in Jordan. SN Soc Sci.

[19] Bao A, Colby DJ, Trang T (2016). Correlates of HIV testing among transgender women in Ho Chi Minh, Vietnam. AIDS Behav.

[20] de Villiers L, Thomas A, Jivan D (2020). Stigma and HIV service access among transfeminine and gender diverse women in South Africa - a narrative analysis of longitudinal qualitative data from the HPTN 071 (PopART) trial. BMC Public Health.

[21] Frisell T, Lichenstein P, Rahman Q, Langstrom N (2010). Psychiatric morbidity associated with same-sex sexual behaviour: influence of minority stress and familial factors. Psychol Med.

[22] Logie CH, James L, Tharao W, Loutfy MR (2011). HIV, gender, race, sexual orientation, and sex work: a qualitative study of intersectional stigma experienced by HIV-positive women in Ontario, Canada. PLoS Med.

[23] Ganju D, Saggurti N. Stigma, violence and HIV vulnerability among transgender persons in sex work in Maharashtra, India. Cult Health Sex. 2017 Aug;19(8):903–17. 10.1080/13691058.2016.1271141. 10.1080/13691058.2016.1271141 PMC6176758 28132601

[24] Hall A, Joseph O, Devlin S (2021). That same stigma… that same hatred and negativity:”a qualitative study to understand stigma and medical mistrust experienced by people living with HIV diagnosed with COVID-19. BMC Infect Dis.

[25] Stringer KL, Marotta P, Baker E (2019). Substance use stigma and antiretroviral therapy adherence among a drug-using population living with HIV. AIDS Patient Care STDs.

[26] Stangl AL, Lloyd JK, Brady LM, Holland CE, Baral S (2013). A systematic review of interventions to reduce HIV-related stigma and discrimination from 2002 to 2013: how far have we come?. J Int AIDS Soc.

[27] Li M, Long J, Wang X et al. A Comparison of COVID-19 Stigma and AIDS Stigma During the COVID-19 Pandemic: A Cross-Sectional Study in China. Front Psychiatry. 2021 Dec 1;12:782501. 10.3389/fpsyt.2021.782501. 10.3389/fpsyt.2021.782501 PMC8671734 34925108

[28] Hall A, Joseph O, Devlin S (2021). That same stigma… that same hatred and negativity:”a qualitative study to understand stigma and medical mistrust experienced by people living with HIV diagnosed with COVID-19. BMC Infect Dis.

[29] Shoib S, Ullah I, Ori D, Saleem SM, Hashmi N, Islam SMS. COVID-19, stigma and mental health: roots and solutions. Rev Colomb Psiquiatr. 2021 Nov;9. 10.1016/j.rcp.2021.10.004. 10.1016/j.rcp.2021.10.004 PMC8576097 34776544

[30] Rueda S, Mitra S, Chen S et al. Examining the associations between HIV-related stigma and health outcomes in people living with HIV/AIDS: a series of meta-analyses. BMJ Open 2016 Jul 13;6(7):e011453. 10.1136/bmjopen-2016-011453. 10.1136/bmjopen-2016-011453 PMC4947735 27412106

[31] Pescosolido BA, Martin JK, Lang A, Olafsdottir S (2008). Rethinking theoretical approaches to stigma: a Framework integrating normative influences on Stigma (FINIS). Soc Sci Med.

[32] Stangl AL, Earnshaw VA, Logie CH (2019). The health stigma and discrimination Framework: a global, crosscutting framework to inform research, intervention development, and policy on health-related stigmas. BMC Med.

[33] Pescosolido BA (1992). Beyond rational choice: the social dynamics of how people seek help. Am J Sociol.

[34] Ssentongo P, Heilbrunn ES, Ssentongo AE (2021). Epidemiology and outcomes of COVID-19 in HIV-infected individuals: a systematic review and meta-analysis. Sci Rep.

[35] Good to read (https://link.springer.com/article/10.1007/s11904-021-00593-8)

[36] Aponte-Melendez Y, Mateu-Gelabert P, Fong C, Eckhardt B, Kapadia S, Marks K (2021). The impact of COVID-19 on people who inject drugs in New York City: increased risk and decreased access to services. Harm Reduct J.

[37] Benoit C, Ouellet N, Jansson M (2016). Unmet health care needs among sex workers in five census metropolitan areas of Canada. Can J Public Health.

[38] Banerjee D, Nair VS (2020). The untold side of COVID-19: struggle and perspectives of the sexual minorities. J Psychosexual Health.

[39] Shalev N, Scherer M, LaSota ED (2020). Clinical characteristics and outcomes in people living with human immunodeficiency virus hospitalized for coronavirus disease 2019. Clin Infect Dis.

[40] Mirzaei H, McFarland W, Karamouzian M, Sharifi H. COVID-19 among people living with HIV: a systematic review. AIDS Behav. 2021 Jan;25(1):85–92. 10.1007/s10461-020-02983-2. 10.1007/s10461-020-02983-2 PMC7391049 32734438

[41] Tesoriero JM, Swain CE, Pierce JL (2021). COVID-19 outcomes among persons living with or without diagnosed HIV infection in New York State. JAMA Netw Open.

[42] Favara G, Barchitta M, Maugeri A, Faro G, Agodi A. HIV infection does not affect the risk of death of COVID-19 patients: a systematic review and meta-analysis of epidemiological studies. J global health. 2022;12. 10.7189/jogh.12.05036 PMC9380965 35972980

[43] Riggle ED, Drabble LA, Bochicchio LA, Wootton AR, Veldhuis CB, Munroe C, Hughes TL (2021). Experiences of the COVID-19 pandemic among african american, latinx, and White sexual minority women: a descriptive phenomenological study. Psychol Sex Orientat Gend Divers.

[44] Schneider A, Conrad D, Pfeiffer A, Elbert T, Kolassa IT, Wilker S (2018). Stigmatization is Associated with increased PTSD risk after traumatic stress and diminished likelihood of spontaneous Remission-A study with East-African Conflict Survivors. Front Psychiatry.

[45] Zandifar A, Badrfam R, Mohammadian Khonsari N, Mohammadi MR, Asayesh H, Qorbani M (2020). Prevalence and Associated factors of posttraumatic stress symptoms and stigma among Health Care Workers in Contact with COVID-19 patients. Iran J Psychiatry.

[46] National Institute of Mental Health. Post-Traumatic Stress Disorder. May 2022. Available at: https://www.nimh.nih.gov/health/topics/post-traumatic-stress-disorder-ptsd. Accessed: 8th November, 2022.

[47] Statistica. Percentage of world population with select mental health disorders as of 2019. https://www.statista.com/statistics/979852/prevalence-of-mental-health-disorders-globally/.

[48] Eng J (2003). Sample size estimation: how many individuals should be studied?. Radiology.

[49] Mirzaei A, Carter SR, Patanwala AE, Schneider CR (2022). Missing data in surveys: key concepts, approaches, and applications. Res Social Administrative Pharm.

[50] Wilson VanVoorhis CR, Morgan BL (2007). Understanding power rules of thumb for determining sample sizes. Tutorials in Quantitative Methods for Psychology.

[51] El Tantawi M, Folayan MO, Nguyen AL, Aly NM, Ezechi O, Uzochukwu BSC (2022). Validation of a COVID-19 mental health and wellness survey questionnaire. BMC Public Health.

[52] Nguyen AL, Brown B, Tantawi ME, Ndembi N, Okeibunor J, Mohammed A, Folayan MO (2021). Time to Scale-up Research Collaborations to address the global impact of COVID-19 - a Commentary. Health Behav Policy Rev.

[53] Ellakany P, Zuñiga RAA, El Tantawi M, Brown B, Aly NM, Ezechi O (2022). Impact of the COVID-19 pandemic on student’ sleep patterns, sexual activity, screen use, and food intake: a global survey. PLoS ONE.

[54] Nzimande NP, El Tantawi M, Zuñiga RAA, Opoku-Sarkodie R, Brown B, Ezechi OC, Uzochukwu BSC, Ellakany P, Aly NM, Nguyen AL, Folayan MO (2022). Sex differences in the experience of COVID-19 post-traumatic stress symptoms by adults in South Africa. BMC Psychiatry.

[55] Folayan MO, Ibigbami O, ElTantawi M, Abeldaño GF, Ara E, Ayanore MA et al. Factors associated with COVID-19 pandemic induced post-traumatic stress symptoms among adults living with and without HIV in Nigeria: a cross-sectional study. BMC Psychiatry. 2022; 22(1):48. 10.1186/s12888-021-03617-0. Erratum in: BMC Psychiatry. 2022;22(1):145. 10.1186/s12888-021-03617-0 PMC8777174 35062920

[56] Tay AK, Rees S, Miah MAA (2019). Functional impairment as a proxy measure indicating high rates of trauma exposure, post-migration living difficulties, common mental disorders, and poor health amongst Rohingya refugees in Malaysia. Transl Psychiatry.

[57] Ruggiero KJ, Del Ben K, Scotti JR, Rabalais AE (2003). Psychometric properties of the PTSD checklist—civilian version. J Trauma Stress.

[58] Weathers FW, Huska JA, Keane TM (1991). PCL-C for DSM-IV.

[59] Kontomanolis EN, Michalopoulos S, Gkasdaris G, Fasoulakis Z (2017). The social stigma of HIV-AIDS: society’s role. HIV AIDS (Auckl).

[60] Li DJ, Chen SL, Yen CF (2019). Multi-dimensional factors associated with illegal substance use among gay and bisexual men in Taiwan. Int J Environ Res Public Health.

[61] Alemu T, Biadgilign S, Deribe K, Escudero HR (2013). Experience of stigma and discrimination and the implications for healthcare seeking behavior among people living with HIV/AIDS in resource-limited setting. Sahara J.

[62] Bhanot D, Singh T, Verma SK, Sharad S (2021). Stigma and discrimination during COVID-19 pandemic. Front Public Health.

[63] World Bank. World Bank country and lending groups. 2020. https://datahelpdesk.worldbank.org/knowledgebase/articles/906519-world-bank-country-and-lending-groups.

[64] Mak WS, Poon CM, Pun LK, Cheung S (2007). Meta-analysis of stigma and mental health. Soc Sci Med.

[65] Hatzenbuehler ML, Phelan JC, Link BG (2013). Stigma as a fundamental cause of population health inequalities. Am J Public Health.

[66] Shaghaghi A, Bhopal RS, Sheikh A (2011). Approaches to recruiting ‘Hard-To-Reach’ populations into re-search: a review of the literature. Health Promot Perspect.

[67] Raifman S, DeVost MA, Digitale JC (2022). Respondent-driven sampling: a Sampling Method for Hard-to-Reach populations and Beyond. Curr Epidemiol Rep.

[68] Abadie R, Habecker P, Carrasco KG (2022). Employing Respondent Driven Sampling (RDS) to recruit people who inject drugs (PWID) and other hard-to-reach populations during COVID-19: Lessons learned. Front Psychiatry.

[69] Pierce M, McManus S, Jessop C (2020). Says who? The significance of sampling in mental health surveys during COVID-19. Lancet Psychiatry.

[70] Hlatshwako TG, Shah SJ, Kosana P et al. Online health survey research during COVID-19. Lancet Digit Health 2021 Feb;3(2):e76–7. 10.1016/S2589-7500(21)00002-9. 10.1016/S2589-7500(21)00002-9 PMC10000261 33509387

[71] Husky MM, Pietrzak RH, Marx BP, Mazure CM. Research on posttraumatic stress disorder in the context of the COVID-19 pandemic: a review of methods and implications in General Population samples. Chronic Stress. 2021;5. 10.1177/24705470211051327. 10.1177/24705470211051327 PMC8576091 34765850

[72] Allen MS, Iliescu D, Greiff S. Single item measures in Psychological Science. Eur J Psychol Assess. 2022;38(1). 10.1027/1015-5759/a000699.

[73] Link BG, Phelan JC (2001). Conceptualizing stigma. Annual Rev Sociol.

[74] Simmons JL (1965). Public stereotypes of deviants. Soc Probl.

[75] Festinger L (1954). A theory of social comparison processes. Hum Relations.

[76] Islam A, Pakrashi D, Vlassopoulos M, Choon L (2021). Stigma and misconceptions in the time of the COVID-19 pandemic?: a field experiment in India. Soc Sci Med.

[77] Krendl AC, Macrae CN, Kelley WM, Fugelsang JA, Heatherton TF (2006). The good, the bad, and the ugly: an fMRI investigation of the functional anatomic correlates of stigma. Soc Neurosci.

[78] Schrimshaw EW, Downing MJ, Cohn DJ (2018). Reasons for non-disclosure of sexual orientation among behaviorally bisexual Men: non-disclosure as Stigma Management. Arch Sex Behav.

[79] Schneider A, Conrad D, Pfeiffer A, Elbert T, Kolassa IT, Wilker S (2018). Stigmatization is Associated with increased PTSD risk after traumatic stress and diminished likelihood of spontaneous Remission-A study with East-African Conflict Survivors. Front Psychiatry.

[80] Blaine B, Crocker J, Baumeister R (1993). Self-esteem and self-serving biases in reactions to positive and negative events: an integrative review. Self-esteem: the puzzle of low self-regard.

[81] Smith RA, Zhu X, Martin MA, Myrick JG, Lennon RP, Small ML, Van Scoy LJ, Data4Action Research Group (2022). Ongitudinal study of an emerging COVID-19 stigma: media exposure, danger appraisal, and stress. Stigma and Health.

[82] Jiang D (2020). Perceived stress and Daily Well-Being during the COVID-19 outbreak: the moderating role of age. Front Psychol.

[83] Miller CT, Major B (2000). Coping with Stigma and Prejudice. The Social psychology of Stigma.

[84] Chandran N, Vinuprasad VG, Sreedevi C, Sathiadevan S, Deepak KS (2022). COVID-19-related Stigma among the affected individuals: a cross-sectional study from Kerala, India. Indian J Psychol Med.

[85] Bhanot D, Singh T, Verma SK, Sharad S (2021). Stigma and discrimination during COVID-19 pandemic. Front Public Health.

[86] Werner P, AboJabel H, Tur-Sinai A (2022). Ageism towards older and younger people in the wake of the COVID-19 outbreak. Maturitas.

[87] Saeed F, Mihan R, Mousavi SZ, Reniers RL, Bateni FS, Alikhani R, Mousavi SB (2020). A narrative review of stigma related to infectious disease outbreaks: what can be learned in the face of the Covid-19 pandemic?. Front Psychiatry.

[88] Bonsaksen T, Heir T, Schou-Bredal I, Ekeberg Ø, Skogstad L, Grimholt TK (2020). Post-traumatic stress disorder and associated factors during the early stage of the COVID-19 pandemic in Norway. Int J Environ Res Public Health.

[89] Vilaplana-Pérez A, Sidorchuk A, Pérez-Vigil A (2020). Assessment of Posttraumatic stress disorder and Educational Achievement in Sweden. JAMA Netw Open.

[90] Tang B, Deng Q, Glik D, Dong J, Zhang L (2017). A Meta-analysis of risk factors for post-traumatic stress disorder (PTSD) in adults and children after earthquakes. Int J Environ Res Public Health.

[91] Brennan JM. Hiding the authentic self: concealment of gender and sexual identity and its consequences for authenticity and psychological well-being. Graduate Student Theses, Dissertations, & Professional Papers. 11789. 2021. https://scholarworks.umt.edu/etd/11789.

[92] Parker R, Aggleton P (2003). HIV and AIDS-related stigma and discrimination: a conceptual framework and implications for action. Soc Sci Med.

[93] Galbadage T, Peterson BM, Awada J (2020). Systematic review and Meta-analysis of sex-specific COVID-19 clinical outcomes. Front Med (Lausanne).

[94] Ditlevsen DN, Elklit A (2010). The combined effect of gender and age on post traumatic stress disorder: do men and women show differences in the lifespan distribution of the disorder?. Ann Gen Psychiatry.

[95] Ascienzo S, Sprang G, Royse D. Gender differences in the PTSD symptoms of polytraumatized youth during isolated phases of trauma-focused cognitive behavioral therapy. Psychological trauma: theory, research, practice, and policy. 2022;14(3):488. 10.1037/tra0001028 33617283

[96] Olff M (2017). Sex and gender differences in post-traumatic stress disorder: an update. Eur J Psychotraumatol.

